# Supplementary material for: PEG-8 Laurate Fermentation of Staphylococcus epidermidis Reduces the Required Dose of Clindamycin Against Cutibacterium acnes
Source: Int J Mol Sci. 2020 Jul 19;21(14):5103. doi: 10.3390/ijms21145103 (PMC7404057; doi:10.3390/ijms21145103)
Supplement: Supplementary file 1 [file ijms-21-05103-s001.pdf]

International Journal of *Molecular Sciences*

MDPI

Supplementary Materials

PEG-8 Laurate Fermentation of *Staphylococcus epidermidis* Reduces the Required Dose of Clindamycin Against *Cutibacterium acnes*

Shinta Marito[1], Sunita Keshari[2], and Chun-Ming Huang[1*]

[1]Department of Biomedical Sciences and Engineering, National Central University, Taoyuan 32001, Taiwan; shintasimbolon53@yahoo.com (S.M); chunming@ncu.edu.tw (C.-M.H)
[2]Department of Life Sciences, National Central University, Taoyuan, 32001, Taiwan; sunitakeshari827@gmail.com (S. K)

*Correspondence: Professor Chun-Ming Huang, Department of Biomedical Sciences and Engineering, National Central University, Taoyuan; E-mail address: chunming@ncu.edu.tw; Tel: +886-3-422-7151 x 36101; Fax: +886-3-425-3427

Received: date; Accepted: date; Published: date

1. Supplementary Information

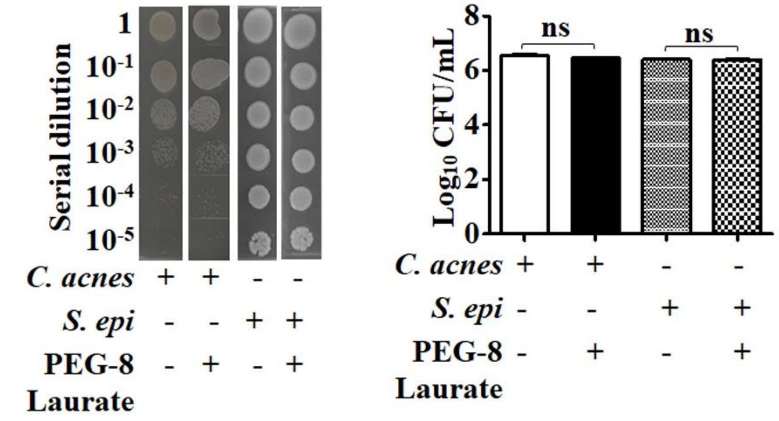

Figure S1. No effect of PEG-8 Laurate on the growth of *C. acnes* or *S. epidermidis*. *C. acnes* or *S. epidermidis* (*S. epi*) (10^5 CFU/mL) was incubated with and without 2% PEG-8 Laurate for 12 h. The CFUs (Log_{10} CFU/mL) were calculated by plating serial dilutions ($1{:}10^0$-$1{:}10^5$) of *C. acnes* or *S. epidermidis* (*S. epi*) on RCM or TSB plates, respectively, 1 day. Data are represented as the mean ± SD of three separate experiments. ns = non-significant.

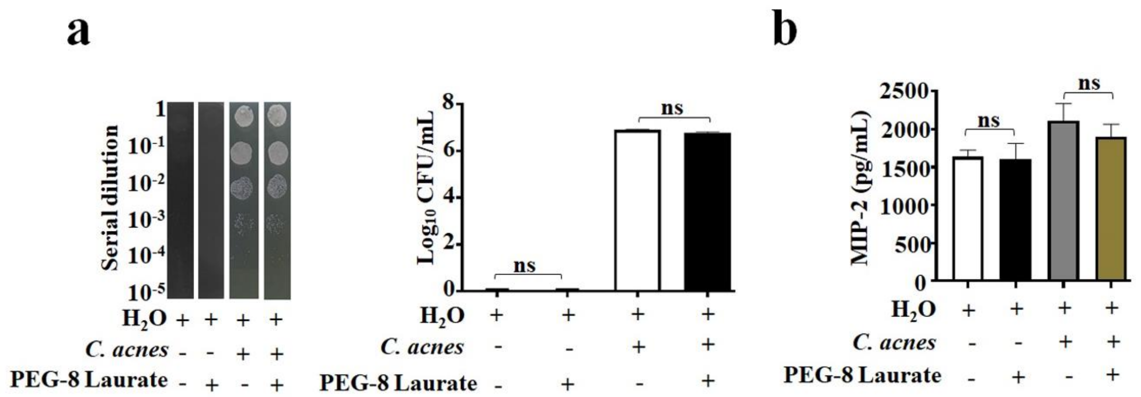

Figure S2. No influence of PEG-8 Laurate on the growth of *C. acnes* and *C. acnes*-induced MIP-2 production. The ears of ICR mice were intradermally injected with *C. acnes* (10^7 CFU) with and without 2% PEG-8 Laurate for 5 d. Mice ears injected with H_2O was included as control. (**a**) the number (Log_{10} CFU) of *C. acnes* in ear homogenates, and (**b**) the levels of MIP-2 cytokines were quantified as described in Materials and Methods. Data are represented as the mean ± SD of three separate experiments using three mice per group; ns = non-significant.

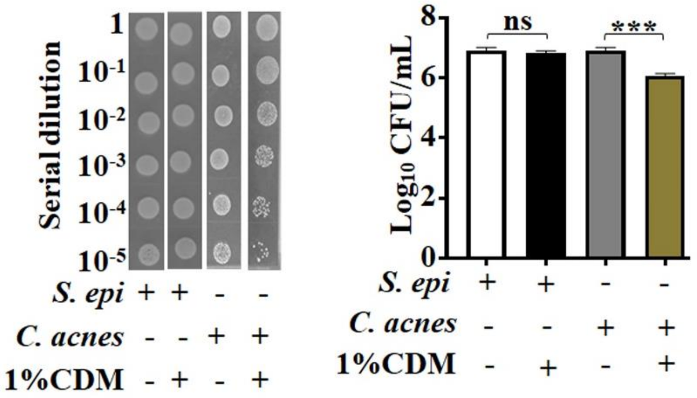

Figure S3. Inhibition of *C. acnes* growth by clindamycin. *S. epidermidis* (*S. epi*) or *C. acnes* (10^7 CFU) was incubated with and without 1% clindamycin (CDM) for 24 h. After plating serial dilution ($1:10^0$-$1:10^5$) of bacterial cultures on agar plates, the CFUs (Log_{10} CFU/mL) of *S. epi* and *C. acnes* were quantified. Data are represented as the mean ± SD, *** $p < 0.001$ (two-tailed t-tests); ns = non-significant.
